# Oridonin induces autophagy via inhibition of glucose metabolism in p53-mutated colorectal cancer cells

**DOI:** 10.1038/cddis.2017.35

**Published:** 2017-02-23

**Authors:** Zhuo Yao, Fuhua Xie, Min Li, Zirui Liang, Wenli Xu, Jianhua Yang, Chang Liu, Hongwangwang Li, Hui Zhou, Liang-Hu Qu

**Affiliations:** 1 Key Laboratory of Gene Engineering of the Ministry of Education, State Key Laboratory of Biocontrol, School of Life Sciences, Sun Yat-Sen University, Guangzhou 510275, China

## Abstract

The Warburg effect is an important characteristic of tumor cells, making it an attractive therapeutic target. Current anticancer drug development strategies predominantly focus on inhibitors of the specific molecular effectors involved in tumor cell proliferation. These drugs or natural compounds, many of which target the Warburg effect and the underlying mechanisms, still need to be characterized. To elucidate the anticancer effects of a natural diterpenoid, oridonin, we first demonstrated the anticancer activity of oridonin both *in vitro* and *in vivo* in colorectal cancer (CRC) cells. Then miRNA profiling of SW480 cells revealed those intracellular signaling related to energy supply was affected by oridonin, suggesting that glucose metabolism is a potential target for CRC therapy. Moreover, our results indicated that oridonin induced metabolic imbalances by significantly inhibiting glucose uptake and reducing lactate export through significantly downregulating the protein levels of GLUT1 and MCT1 *in vitro* and vivo. However, the ATP level in oridonin-treated CRC cells was not decreased when oridonin blocked the glucose supply, indicating that oridonin induced autophagy process, an important ATP source in cancer cells. The observation was then supported by the results of LC3-II detection and transmission electron microscopy analysis, which confirmed the presence of autophagy. Furthermore, p-AMPK was rapidly deactivated following oridonin treatment, resulting in downregulation of GLUT1 and induction of autophagy in the cancer cells. Thus our finding helped to clarify the anticancer mechanisms of oridonin and suggested it could be applied as a glucose metabolism-targeting agent for cancer treatment.

One of the hallmarks of cancer cells is enhanced glucose metabolism and dramatically altered nutrient utilization compared to normal tissues, which was first described by Otto Warburg in the early 1900s^1^ and is now known as the Warburg effect. Glucose is a major source of energy in cancer cells that generates ATP almost through glycolysis, a far less efficient energy generator than mitochondrial respiration.^2^ As the survival of cancer cells predominantly depend on the high rate of glucose consumption and elevated glycolysis, targeting the Warburg effect and glucose metabolism has become an important strategy for cancer therapy. Several factors, including mitochondrial defects, oncogenic stimuli, hypoxia, and aberrantly enhanced expression of glycolytic enzymes, are now considered to be important drivers of the Warburg effect.^2, 3^ Due to their critical role in cancer cells, these glucose metabolism-related proteins and enzymes are believed to be potential targets for drug design and cancer therapy.^4^

There are two main strategies under investigation that target glucose metabolism. One focuses on the regulation of glycolytic flux-related proteins including Gluts, MCTs, lactate dehydrogenase A (LDHA), HK2, and PKM2.^5, 6^ This strategy aims to directly regulate the glucose supply and glycolytic pathways to control the energy production in cancer cells. Another strategy focuses on factors believed to be central for metabolic regulation, including HIF-1a, c-Myc, AKT, mTOR, and AMPK.^7^ These factors control the abundance of proteins that regulate the glucose and energy supply of cancer cells. Several compounds, including WZB117, rapamycin, berberine, and metformin, have been shown to regulate glucose metabolism and exhibit anticancer activities.^8, 9, 10, 11^ However, the mechanisms of anticancer activities by which natural compounds targeting glucose metabolism still need to investigate.

Oridonin is an active diterpenoid isolated from *Rabdosia rubescens* in the 1970s that has potent anti-tumor activities in many types of human cancer both *in vitro* and *in vivo*.^12, 13^ Oridonin treatment had multiple effects such as inhibition of proliferation and induction of cell cycle arrest, apoptotic and autophagic pathways in a variety of cancer cells including those of colorectal carcinoma.^14, 15, 16, 17^ Previous studies have indicated that various factors, including Akt, ERK, FAS, ROS, NF-*κ*B, PI3K, and RTK, were involved in the anticancer activity of oridonin.^17, 18, 19^ Although various mechanisms were proposed to explain the anticancer activity of oridonin, most of the research has been scattered. Oridonin was reported to suppress t(8;21) acute myeloid leukemic (AML) cells via cleavage of the AML1–ETO fusion protein.^20^ Our lab reported that oridonin induced c-MYC proteasomal degradation in chronic myeloid leukemia.^21^ Thus, in contrast to these studies performed in leukemic cells, the underlying mechanisms of oridonin-induced anticancer activity in specific solid tumors and which type of solid tumor shows the best response have not been elucidated, and further studies are needed to provide evidence supporting the use of oridonin as a antineoplastic agent.

To elucidate the mechanism of oridonin in CRC cells, we investigated the miRNA profile by high-throughput sequencing both in the absence and presence of oridonin in CRC cells. These results prompted us to focus on the regulation of the Warburg effect and glucose metabolism. We then confirmed that oridonin affected the cellular glucose supply and lactate exportation via AMPK-related glucose transporter 1 (GLUT1) and monocarboxylate transporter 1 (MCT1) regulation. This mechanism could be a trigger that induces cancer cell autophagy and accelerates cell death. Our findings showed that oridonin could affect glucose metabolism and induce autophagy through a metabolism-related pathway.

## Results

### Oridonin causes cell death through an atypical apoptosis manner in p53-mutated CRC cells and exhibits anticancer activity both *in vitro* and *in vivo*

**Figure 1:**
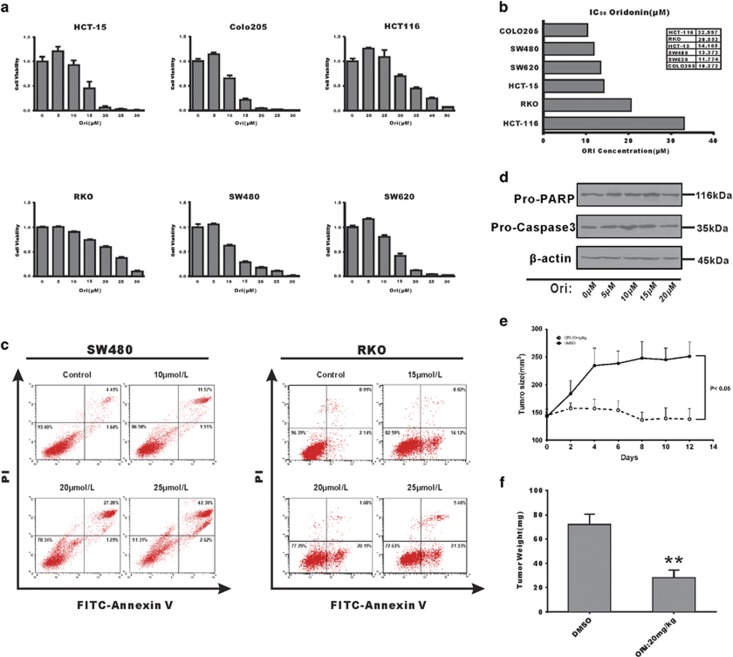
Oridonin inhibits CRC cell proliferation and induces cell death both *in vitro* and *in vivo*. (**a**) Cell viability detected by CCK-8 assays in six CRC cell lines treated with different doses of oridonin for 24 h. (**b**) IC_50_ values of the oridonin in six CRC cells. (**c**) Contour diagrams of flow cytometry analysis of SW480 cells after the 24 h treatment; (**d**), Western blot analysis showing caspases were not activated following oridonin treatment. (**e** and **f**) Oridonin decreased tumor growth in a SW480 xenograft model as determined by assessing both tumor volume and tumor weight

To analyze the *in vitro* effects of the oridonin on colon cancer cells, we treated several cell lines with different doses of oridonin for 24 h and assessed the effects on proliferation and survival. Significant cytotoxic activity was observed in all six CRC cell lines (HCT-15, COLO205, HCT116, RKO, SW480, and SW620) in a dose-dependent manner, with IC_50_ values ranging between 10 and 32 *μ*M at 24 h (Figures 1 a and b). Then, SW480 and RKO cells were analyzed by flow cytometry following Annexin V-fluorescein isothiocyanate (FITC) and propidium iodide (PI) dual labeling after the 24 h oridonin treatment. Results indicated that cell death rate is upregulated with oridonin concentration in both SW480 and RKO cells, but no typical phenotype of apoptosis was observed in p53-mutated CRC cell SW480, similar results were also detected in another p53-mutated CRC cell HCT-15 (Supplementary Figure 1), whereas in p53 wild-type (WT) CRC cell RKO showed classical apoptosis (Figure 1c). To confirm these results, we performed Western blots to measure the protein levels of caspase 3 and PARP, the most important molecular biomarkers of apoptosis. The results indicated that both caspase 3 and PARP were not significantly activated, and oridonin did not significantly induce apoptosis in SW480 cells (Figure 1d). But clearly caspase 3 activation and PARP cleavage were detected in RKO cells under oridonin treatment (Supplementary Figure 2). Animal xenograft studies indicated that tumor growth significantly delayed in nude mice treated with oridonin, and tumor weight was also significantly decreased compared with that of the solvent control group (Figures 1e and f, *P*<0.05), whereas the body weight changes showed no apparent differences between the treatment and control groups (data not shown). Collectively, these results indicated that oridonin exhibits potent anticancer effects on CRC cells both *in vitro* and *in vivo*, and it induced cell death through an atypical form of apoptosis in p53-mutated CRC cells.

### Cellular energy homeostasis is altered by oridonin

**Figure 2:**
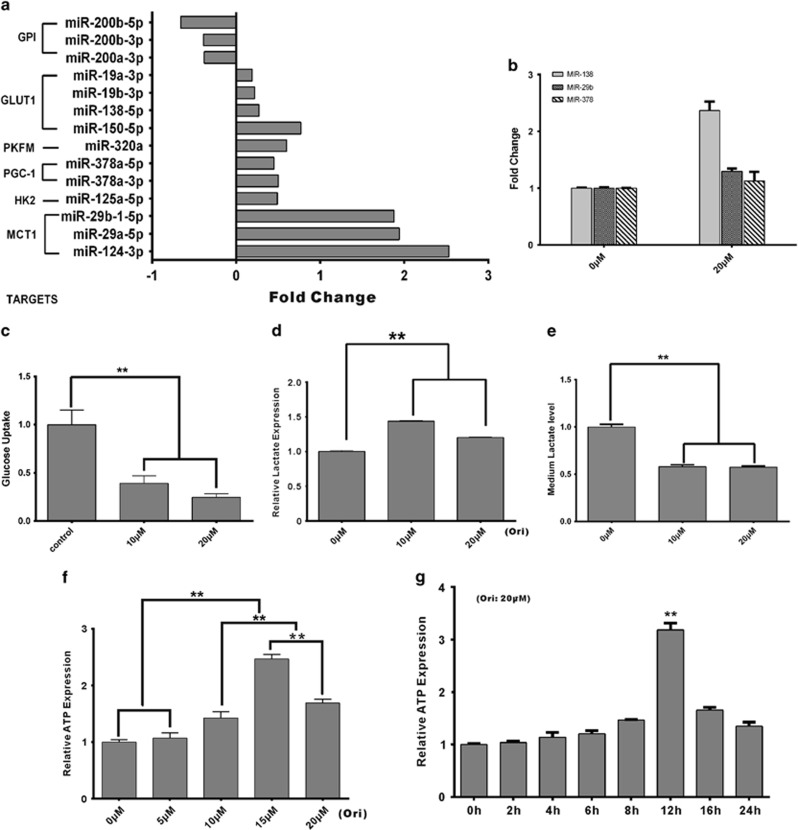
Oridonin affects intracellular energy homeostasis. (**a**) A histogram showing differential expression of miRNAs related to glucose metabolism following oridonin treatment. (**b**) Verification of miRNAs by qPCR analysis. (**c**) Glucose uptake was inhibited by oridonin in SW480 cells. (**d** and **e**) Oridonin inhibited intracellular lactate export after a 24 h oridonin treatment. (**f**) Intracellular ATP levels were upregulated after the 24 h oridonin treatment. (**g**) ATP analysis during a time course experiment. ATP was significantly upregulated after a 12 h oridonin treatment. (**P<0.05)

To investigate the anticancer mechanisms of oridonin, we performed high-throughput sequencing to analyze miRNA expression, as miRNA expression profiling can be used to assess the responses to cancer therapies.^21^ The expression of 14 miRNAs reported to regulate key enzymes of the Warburg effect^22^ was significantly changed upon oridonin treatment in the SW480 cell line (Figure 2a). The results showed that MCT1-related miRNAs (miR-124-3p, miR-29b-1-5p, and miR-29a-5p) had the most significant expression changes, with more than two-fold increases, and the *GLUT1* gene had the greatest number of related miRNAs (miR-130b-5p, miR-532-5p, miR-138-5p, miR-150-5p, miR-19b-3p, and miR-19a-3p) that were altered following oridonin treatment. Other miRNAs, including miR-378a-3p/5p targeting PGC-1, miR-200a/b/c-3p and miR-200b-5p targeting GPI, miR-125a-5p targeting HK2, and miR-320a targeting PKFM, also showed expression changes after oridonin treatment. Then, the expression of several representative miRNAs was confirmed by qRT-PCR (Figure 2b). From these results we can hypothesis that oridonin may affect cancer cell metabolism through those proteins involved in the Warburg effect.

To confirm this hypothesis, we investigated the intracellular energy status to assess the relationships between oridonin and cancer cells. First we evaluated the differences in glucose uptake, cellular lactate rate, and ATP level upon oridonin treatment in CRC cells. Glucose consumption of the SW480 cells significantly decreased after oridonin stimulation (Figure 2c). Furthermore, intracellular lactate concentration was upregulated, but extracellular lactate decreased in SW480 cells after 24 h of oridonin treatment suggesting lactate exportation is inhibited (Figures 2d and e). Intracellular ATP levels significantly increased by more than two-fold after the 24 h oridonin treatment in a dose-dependent manner (Figure 2f). To further elucidate how the ATP level increases, ATP levels were examined under 20 *μ*M oridonin treatment in different times (0 h, 2 h, 4 h, 6 h, 8 h, 12 h, 16 h, and 24 h). The ATP level was not significantly different at these time points, except the 12 h time point, which showed a substantial ATP increase of more than three-fold (Figure 2g). Overall, these results suggested that oridonin could reduce glucose uptake, inhibit lactate export, and influence the cellular ATP supply to regulating cellular energy homeostasis.

### Oridonin regulates cellular energy flow through GLUT1 and MCT1

**Figure 3:**
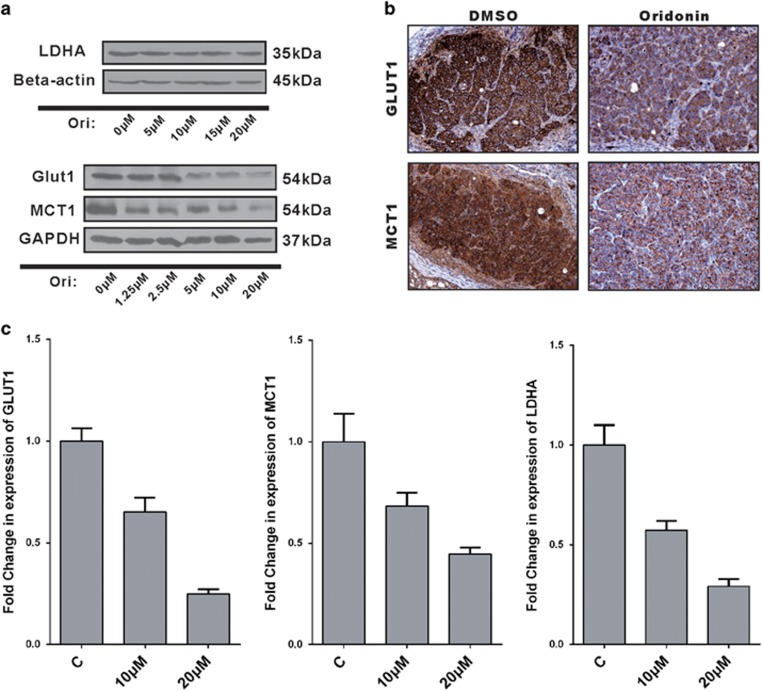
Oridonin decreased the expression of glycolysis-related proteins. (**a**) Oridonin did not affect the expression of LDHA but caused a dose-dependent decrease of GLUT1 and MCT1 following 24 h oridonin treatment. (**b**) Immunohistochemistry analysis of GLUT1 and MCT1 in SW480 xenograft tumors. The sections were developed by diaminobenzidine and counterstained with hematoxylin. (**c**) Oridonin downregulated mRNA expression of glut1, mct1, and ldha in SW480 cells

The above findings suggesting us that some of the functional proteins and enzymes that regulate these processes in glycolytic metabolism were affected by oridonin. To verify this hypothesis, we first analyzed various proteins, including the GLUT1 and MCT1, which have been reported to be major regulators of the glucose supply and lactate export in colorectal carcinoma.^23, 24^ The results showed that the GLUT1 and MCT1 protein levels were downregulated following oridonin treatment for 24 h in a dose-dependent manner (Figure 3a). However, the protein level of LDHA, which is a crucial enzyme in controlling glycolysis, did not show similar results to those of GLUT1 and MCT1 (Figure 3a). Then, mRNA expression of these proteins was analyzed by qRT-PCR, and the results indicated that oridonin downregulated the mRNA expression of GLUT1, MCT1, and LDHA (Figure 3c). In addition, SW480 xenograft tumors treated with oridonin showed substantially reduced levels of GLUT1 and MCT1 compared with those of the control group, which confirmed the *in vitro* results (Figure 3b). Taken together, the data showed that oridonin affected glucose metabolism through the regulation of an energy supplier (GLUT1) and waste exporter (MCT1) but not the rate-limiting enzyme LDHA, which controls glycolytic flux.

### Oridonin induces autophagy through deactivation of AMPK and downregulation of GLUT1 in SW480 cells

The above observations indicated that the intracellular ATP level was upregulated after a 24 h oridonin treatment, and the time course showed that ATP was upregulated at only a few time points during oridonin treatment. This result was unexpected as we had demonstrated that oridonin could downregulate the GLUT1 and MCT1 expression to reduce glucose consumption and decrease the lactate production of the cell, which indicating intracellular ATP level should decrease after oridonin treatment. There are four ATP sources in normal cells, including oxidative phosphorylation, glycolysis, glutaminolysis, and autophagy.^25^ The phenotype of the cancer cells indicated that oxidative phosphorylation and glutaminolysis cannot be the sources of the high intracellular ATP levels. Our finding of glucose uptake inhibition also suggested that glycolysis was not responsible for the ATP upregulation. Thus, we excluded three of the ATP sources and hypothesized that oridonin treatment induced cancer cell autophagy, resulting in intracellular ATP upregulation in SW480 cells.

**Figure 4:**
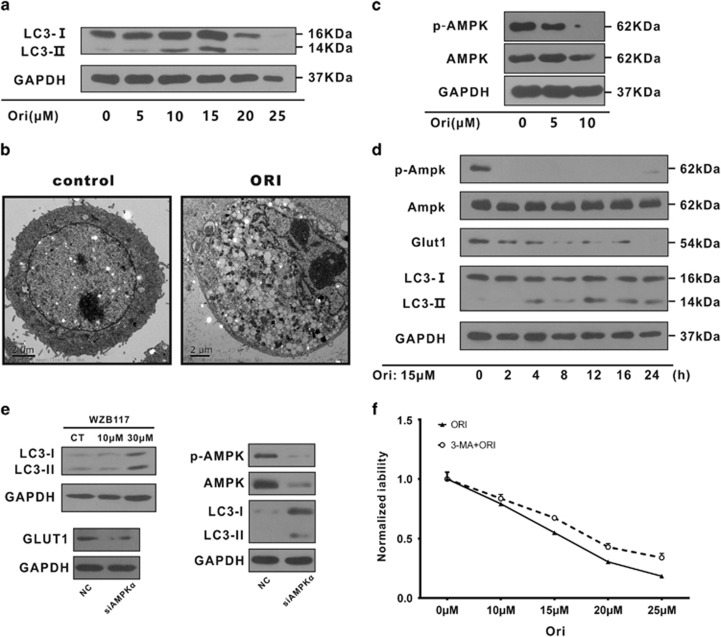
Oridonin could induce autophagy in a GLUT1-related but AMPK activation-independent manner. (**a**) Oridonin activated the LC3-II expression in SW480 cells after a 24 h treatment. (**b**) The ultrastructure of SW480 cells was observed by electron microscopy after oridonin treatment. Clear autophagosomes were observed in the oridonin treatment group, whereas this was not found in the control group. (**c**) AMPK was deactivated by oridonin as there was a decrease in p-AMPK in SW480 cells. (**d**) Detection of these proteins in a time course experiment after oridonin treatment. Oridonin downregulated p-AMPK and GLUT1 in a time-dependent manner in SW480 cells. (**e**) The autophagy inhibitor 3-MA (5 mmol/l) could alleviate the cytotoxicity of oridonin in SW480 cells. (**f**) Blocking AMPK by siRNA downregulated GLUT1 and induced LC3-II expression

To confirm our hypothesis, we first analyzed the expression of LC3, the cleaved and lipidated form (LC3-II) of which is widely regarded as an autophagosome marker. As shown in Figure 4a, the expression of LC3-II was upregulated following oridonin treatment in a dose-dependent manner. Then, transmission electron microscopy analysis was employed for a more detailed examination of the autophagy. Clear morphological characteristics of autophagosomes were observed in oridonin-treated SW480 cells, but no such phenomena were observed in the control group (Figure 4b). Some other autophagy-related proteins including beclin-1 and Atg5 could also by upregulated by oridonin treatment in HCT-15 cells, whereas oridonin could not induce those proteins in HCT116 cells (Supplementary Figure 3). In nude mice, oridonin could also upregulate the LC3-II expression in SW480 cells, accordingly (Supplementary Figure 4). These results verified our hypothesis that oridonin induced autophagy in p53 mutant type (MT) cells.

AMPK, an important energy regulator in the cell that controls the expression of GLUT1 and MCT1, and promotes autophagy were detected. p-AMPK levels were substantially decreased with oridonin treatment, but total AMPK protein was unchanged (Figure 4c). Then, a time course experiment was carried out to assess the effects over time, and the results showed that oridonin could rapidly and significantly deactivate p-AMPK in <2 h, GLUT1 expression decreased in <4 h, whereas LC3-II levels were upregulated (Figure 4d). The LC3-II level also upregulated when we blocked GLUT1 with a specific inhibitor, WZB117, whereas similar results were observed when we knockdown AMPK expression, accompanied by a reduction in the GLUT1 level (Figure 4e). To further confirm oridonin-induced cell death via autophagy, by using a autophagy inhibitor 3-MA to block this process, we analyzed cell viability to confirm the autophagy in oridonin-induced cell death. The results revealed that 3-MA could alleviate the viability inhibition by oridonin treatment (Figure 4f).

Collectively, these results suggest that oridonin induced autophagy through downregulation of AMPK-GLUT1 and caused cell death in colorectal cancer cells.

## Discussion

Over the past decade, the mechanisms underlying the anti-tumor activities of oridonin have been widely studied to provide support for the use of oridonin in clinical cancer therapy.^21, 26, 27, 28, 29^ Most studies, however, have not elucidated the detailed functions, mechanisms, and signaling pathways of its anticancer effect on solid tumors. Therefore, we conducted the present study to clarify the possible relationships between CRC cells and oridonin, and to explore the potential mechanisms of the anti-tumor activities of oridonin.

The cytotoxic activity and apoptosis induction of oridonin have been reported in several CRC cell lines including HT-29, HCT116, SW480, and SW620.^16, 28, 30, 31, 32^ But a very interesting finding grabbed our attention that although clear anti-proliferative activity was observed both *in vitro* and vivo, no typical apoptosis phenomenon was observed in SW480 cells, whereas clear apoptosis was detected in RKO cells using flow cytometry with Annexin V-FITC/PI labeling (Figure 1c). Thus, we hypothesized that oridonin caused cell death via a distinct process that was different from normal apoptosis in SW480 cells. Apoptosis biomarker analysis CRC cells (Figure 1d and Supplementary Figure 2) supported our hypothesis. These results were unexpected as apoptosis is considered to be the primary mechanism of oridonin anticancer activity, similar to what was observed in RKO cells and some previous reports.^21, 28^ There are three major types of morphologically distinct cell death: apoptosis (type I cell death), autophagic cell death (type II), and necrosis (type III).^33^ Here we did not observe the loss of the plasma membrane, which is characteristic of necrotic cell death. Thus, we could exclude both type I and type III cell death. Then, autophagic cell death could be the key mechanism underlying oridonin-induced cell death in SW480 and HCT-15 cells. This hypothesis was also supported by the results of our autophagy analysis. The results obtained in RKO and HCT116 cells suggested that oridonin promotes anti-tumor activity in both apoptotic and non-apoptotic manners. Previous studies have found that WT P53 protein was expressed in RKO and HCT116 cells, whereas SW480 and HCT-15 cells only expressed the MT P53 proteins. Studies indicated that CRC cells with MT P53 were more chemo-resistant than those with WT p53 due to their poor ability to activate apoptosis and initiation of apoptosis is the most important functions of P53.^34, 35^ Thus, we hypothesized that oridonin preferentially induced cell death through apoptosis in WT P53 cancer cells, whereas in those MT P53 cancer cells oridonin triggered another mechanism (e.g., autophagy) to compensate for the low apoptotic activity. Our study in those CRC cells supported our hypothesis, but further research is still needed to conduct the analysis in a more comprehensive way.

Accumulating evidence suggested that miRNA expression profiling could be used to elucidate various processes, such as apoptosis, autophagy,^36^ immunity,^37^ and cellular metabolism,^22, 38, 39^ in response to therapy in human cancers. Combining our data from high-throughput sequencing with the data reported in previous studies suggests that oridonin may influence the Warburg effect and affect its role through a metabolism-regulated pathway. A key finding in our study is that oridonin could decrease the glucose uptake and induce intracellular lactate accumulation (Figures 2c and d). Thus, we concluded that oridonin blocked the metabolic flux and inhibited anaerobic glycolysis, affecting the Warburg effect of the cancer cells. The Warburg effect describes the high flux of glucose through glycolysis, which converts hexoses to trioses, and results in pyruvate which converted to lactate.^40^ This whole reaction proceeds in the cytoplasm to produce a net 2 ATP from each glucose molecule, which is far less than that of the Krebs cycle, which generates 36 or 38 ATP. Due to the low efficiency of energy generation, cancer cells rely on the high rate of glucose consumption and lactate exportation to maintain the cellular energy supply and homeostasis. Targeting glucose metabolism of cancer cell has been shown to be a successful therapeutic strategy.^41^ This strategy not only altered cancer initiation and progression but also improved existing approaches, including diagnostic, drug resistance, or side effect reduction strategies, and is now a popular topic in cancer research.^5, 41, 42^ This study is the first to show that oridonin could target glucose metabolism, and it increases our knowledge of the effects of oridonin.

While it is clear that oridonin could affect intracellular energy flux, the underlying mechanism of how oridonin targets glucose metabolism is still under investigation. Various strategies aimed at regulating glucose metabolism are being researched and generally focus on three steps: 1. targeting glucose transport (e.g., Gluts) to control the cellular energy supply; 2. targeting rate-limiting enzymes (e.g., HK2, PKM2) to influence the glycolytic pathway; and 3. targeting lactate generation and export (e.g., MCTs, LDHA) to block the final stage of glycolysis. Our data showed that oridonin significantly downregulated GLUT1 and MCT1 expression in CRC cells. GLUT1 is one of the most important regulators of glucose import, shows variable levels in many tissues and is believed to be responsible for basal glucose uptake,^24^ whereas MCT1 plays a major role in transport of lactate, pyruvate, and other short-chain monocarboxylates across the membrane in a freely reversible manner.^43^ Thus, our results indicated that oridonin can block both energy supply and lactate export through the regulation of GLUT1 and MCT1 in CRC cells. Both GLUT1 and MCT1 expression are essentially ubiquitous across all mammalian tissues at low levels, whereas in most cancer cells, they are highly expressed.^2, 44^ The overexpression of GLUT1 is correlated to hypoxia, poor prognosis, and drug resistance in CRC.^24, 45, 46, 47^ Other reports found a similar relationship between MCT1 and various CRC characteristics including drug resistance, poor prognosis, and hypoxia.^48, 49^ However, the most important biological functions of these two proteins are to supply carbohydrates and export the waste lactate to maintain cellular energy homeostasis.^50, 51, 52^

The ATP increase observed after oridonin treatment (Figure 2f) was unexpected, as we had demonstrated that oridonin could block glucose uptake and lactate export. We predicted that the ATP level would decrease along with the reduced glucose levels. There are four major ATP supply methods – oxidative phosphorylation, glutaminolysis, autophagy, and glycolysis.^25, 53^ Combining our results shown in Figure 2c and the essential characteristics of cancer cells, we concluded that because cancer cells only maintain low levels of oxidative phosphorylation, glycolysis was blocked by the decrease in glucose, and glutaminolysis can only produce low levels of ATP, and autophagy was the likely mechanism of ATP upregulation caused by oridonin treatment. The results of autophagy-related biomarker analysis and transmission electron microscopy analysis confirmed the hypothesis (Figure 4; Supplementary Figure 3) that autophagy is induced by oridonin in p53-mutated CRC cells. In addition, this was supported by the results of flow cytometry analysis (Figure 1c; Supplementary Figure 1). Although induction of autophagy by oridonin has been reported for years in several cell lines, this is the first time a similar phenomenon has been reported in CRC cell lines.

Glucose metabolic disorders are intimately related to cellular autophagy, glucose deprivation, and decreased cellular levels of ATP, leading to activation of autophagy.^54^ To determine exactly how oridonin triggers autophagy, we analyzed ATP levels and related proteins in a time course experiment. The ATP levels only increased (Figure 2g) for a few hours after the detection of LC3-II activation (Figure 4d), which is a marker of autophagy. Thus, we concluded that autophagy is responsible for the ATP upregulation. Furthermore, a reduction in GLUT1 accompanied the activation of LC3-II (Figure 4d), suggesting that GLUT1 was correlated with the activation of autophagy. This is consistent with a recent report indicating that downregulation of GLUT1 was an important trigger of autophagy.^55^ Interestingly, it has been reported that activation of AMPK could promote autophagy.^56^ However, in our study, the rapid deactivation of AMPK indicated that oridonin induced autophagy independent of AMPK activation. As AMPK is upstream of GLUT1 and MCT1,^51, 57^ the depletion of p-AMPK contributed to the oridonin-induced downregulation of GLUT1 and MCT1. In addition, a recent report indicated that AMPK inhibition could also be a trigger to activate autophagy,^58^ which supports our conclusion that oridonin induces autophagy through AMPK deactivation-mediated GLUT1 downregulation, as shown in Figure 4f. Autophagy is an important process in response to cellular stress and generally acts as a pro-survival mechanism for the maintenance of cellular homeostasis following environmental stimuli, such as endoplasmic reticulum stress or hypoxic stress.^59, 60^ Inhibition of autophagy reduced the oridonin-induced anti-proliferative activity (Figure 4e), indicating that the autophagy induced by oridonin did not typically promote cell survival but instead a mechanism called autophagy-mediated cell death (which could involve a standard mechanism of cell death, such as apoptosis, but is triggered by autophagy).^61^

**Figure 5:**
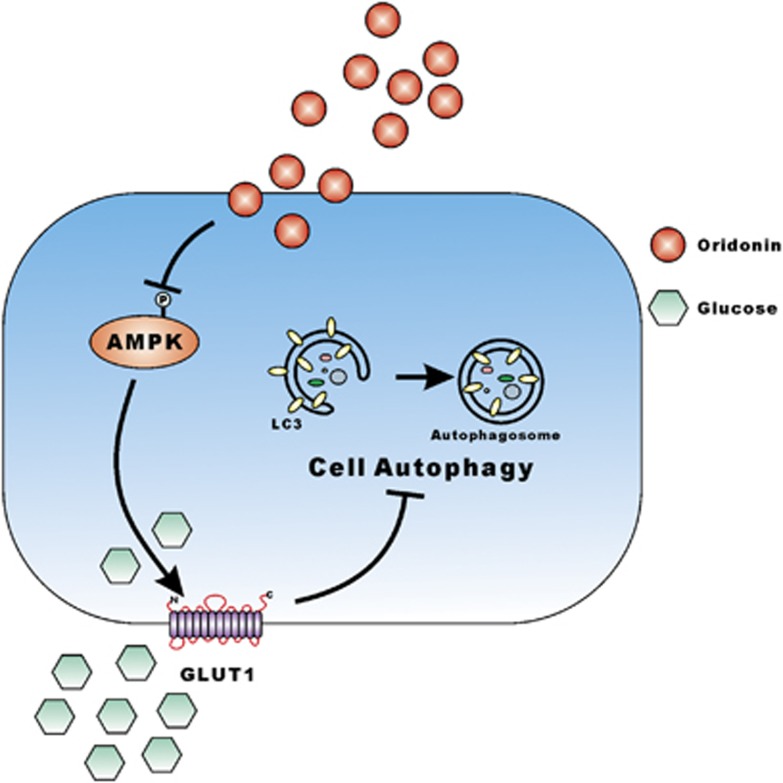
The proposed molecular mechanisms of action of oridonin. Oridonin strongly inhibited p-AMPK in a very short time period and then downregulated glut1 expression, and inhibited glucose uptake of the cancer cells. The glucose absorption was affected and induced cancer cell autophagy

In summary, the results of these studies support the hypothesis that oridonin altered intracellular energy homeostasis and induced autophagy. We demonstrated the potent anticancer activity of oridonin *in vitro* and *in vivo* in CRC cells and determined that the underlying mechanism of oridonin involves induction of metabolic disorders and autophagy. Oridonin significantly downregulated GLUT1 and MCT1, and altered energy homeostasis in cancer cells. Meanwhile, deactivation of AMPK induced GLUT1 downregulation, and metabolic disorders were related to oridonin-mediated autophagy. Moreover, our data suggested that autophagy is associated with the anti-tumor activity induced by oridonin in CRC. These mechanisms are summarized in Figure 5.

## Materials and methods

### Cell lines and cell cultures

All six CRC cell lines (HCT-15, COLO205, HCT116, RKO, SW480, and SW620) were obtained from the cell bank of the Chinese Academy of Sciences (Shanghai, China). Cells were cultured in RPMI 1640 or DMEM containing 10% fetal bovine serum (HyClone, SV30160, Logan, UT, USA), 100 U/ml penicillin, and 100 mg/ml streptomycin in a humidified atmosphere containing 5% CO_2_ at 37 °C.

### Cell viability and apoptosis assays

The cytotoxicity of oridonin in the CRC cells was assessed using a Cell Counting Kit-8 (Dojindo, Laboratories, Kumamoto, Japan). All CRC cells were seeded in 96-well microplates with appropriate numbers overnight and were then incubated at 37 °C with oridonin for 24 h. The optical density (OD) was measured by an ELISA reader at 450 and 630 nm. The percentage of cytotoxicity was calculated by the following equation: Cytotoxicity (%)=(OD control group × OD treatment group)/OD control group × 100%.

A FACS Calibur flow cytometer (BD Biosciences, San Jose, CA, USA) was used to analyze apoptosis with FITC-Annexin V/PI double staining. The data were analyzed by FlowJo software (TreeStar, Ashland, OR, USA).

### Measurement of intracellular ATP and l-lactate levels

Intracellular ATP was measured using CellTiter-Glo Reagent (Promega, Madison, WI, USA). Briefly, cells were harvested, quantified, and then lysed with lysis buffer. Fifty microliters from each diluted sample was mixed with an equal volume of CellTiter-Glo Reagent, and the sample was incubated at room temperature for 10 min and analyzed using a GloMax luminometer (GloMax 96 Microplate Luminometer; Promega).

After oridonin treatment, the medium was removed from the cells, and lactate levels in the extracellular medium were measured using a l-lactate Colorimetric Assay Kit (Abcam, Cambridge, UK). Then, the intracellular lactate levels were measured in the cell lysates. Data were normalized to the final cell counts.

### RNA extraction and real-time quantitative PCR

Total RNA was extracted using the TRIzol reagent (Invitrogen, CA, USA). First-strand cDNA was synthesized with Prime Script RT Master Mix (TaKaRa, Dalian, China). Then, quantitative PCR was conducted using SYBR Green PCR Master mix (TaKaRa) on a Bio-Rad Real-Time PCR instrument (Bio-Rad, CA, USA). The sequences of the primers were as follows (in the 5′ to 3′ orientation): GLUT1 forward, 5′-CTT TGT GGC CTT CTT TGA AGT-3′; GLUT1 reverse, 5′-CCA CAC AGT TGC TCC ACA T-3′; MCT1 forward, 5′-GGT GTCATT GGA GGT CTT GGG-3′ MCT1 reverse, 5′-GGC CAA TGG TCGCTTCTT G-3′; GAPDH forward, 5′-TGA CCT GCC GTC TAG AAA AAC C-3′ GAPDH reverse, 5′-GCC AAA TTC GTT GTC ATA CCA GG-3′; LDHA forward, 5′-ATCTTG ACC TAC GTG GCT TGG A-3′; LDHA reverse, 5′-CCA TAC AGG CAC ACTGGA ATC TC-3′. The data were analyzed by StepOne software (Applied Biosystems, Waltham, MA, USA) with GAPDH as the constitutive marker.

### Western blot

Cells were rinsed twice with cold PBS and homogenized with RIPA lysis buffer (Cell Signaling Technology, Beverly, MA, USA), and a protease inhibitor cocktail (Roche, Berlin, Germany) was added according to the manufacturer's recommendation (40 *μ*g per sample). Proteins were separated by 10% SDS-PAGE and electrophoretically transferred onto a polyvinylidene fluoride membrane (Millipore, Temecula, CA, USA). The membrane was washed with TBST, blocked with 5% skimmed milk, diluted in TBST for 1 h and then incubated with appropriate primary antibodies (anti-p-AMPK, GAPDH, caspase 3, PARP, LDHA, LC3, and *β*-actin obtained from Cell Signaling Technology, anti-GLUT1 and MCT1 obtained from Millipore, and all the antibodies were diluted 1:1000) overnight at 4 °C. The membranes were then washed for 5 min three times with TBST, and primary antibodies were detected with goat anti-rabbit IgG (CST) antibodies conjugated to horseradish peroxide. Bands were visualized with the ECL Western Blot Detection System (CST) and subjected to autoradiography using X-ray film.

### Animal models

A SW480 xenograft-bearing mouse model was constructed using 6-week-old BALB/c nude mice (Sun Yat-sen University). Each mouse was subcutaneously injected with 6 × 10^6^ SW480 cells. When the volume of the tumors reached 140 mm^3^, the mice were randomly assigned to two groups and treated intraperitoneally with oridonin (15 mg/kg) or vehicle (2% DMSO). All mice were killed after a 12-day treatment, and the tumor volumes (length × width^2^ × 0.5236) and body weights were determined. Then, the tumors were collected for immunohistochemistry analysis.

### Transmission electron microscopy analysis

Cells were washed with PBS and fixed in 3% glutaraldehyde dissolved in 0.1 mol/l phosphate buffer for 30 min at room temperature. The cells were then postfixed for 1 h in 1% OsO_4_ (Sigma-Aldrich, St. Louis, MO, USA). After dehydration, the cells were embedded in Epon 812 (SPI Supplies, West Chester, PA, USA) and polymerized at 60 °C for 24 h. EM images were acquired from thin sections using a JEM1400 electron microscope (JEOL, Tokyo, Japan). Images were digitally acquired from a randomly selected pool under each condition.

### Statistical analyses

The results are expressed as the mean±S.D. Comparisons were made to determine significant differences between individual groups by ANOVA. All tests were performed using SPSS 19 (SPSS Inc., Chicago, IL, USA), and *P*<0.05 was considered significant. All experiments were performed at least three times.
