# Supplementary material for: Oridonin induces autophagy via inhibition of glucose metabolism in p53-mutated colorectal cancer cells
Source: Cell Death Dis. 2017 Feb 23;8(2):e2633–. doi: 10.1038/cddis.2017.35 (PMC5386482; doi:10.1038/cddis.2017.35)
Supplement: Supplementary Information [file cddis201735x1.docx]

**Summary of Supplementary Files content**

- Supplementary_information: Contains Supplementary Figure Legends
- Supplementary Figure 1.
- Supplementary Figure 2.
- Supplementary Figure 3.
- Supplementary Figure 4.
