# Supplementary material for: Oridonin induces autophagy via inhibition of glucose metabolism in p53-mutated colorectal cancer cells
Source: Cell Death Dis. 2017 Feb 23;8(2):e2633–. doi: 10.1038/cddis.2017.35 (PMC5386482; doi:10.1038/cddis.2017.35)
Supplement: Supplementary Information [file cddis201735x2.docx]

**Supplementary Figure 1:** Oridonin induce HCT-15 cell death through an atypical apoptosis manner. Contour diagrams of flow cytometry analysis of HCT-15 cells after 24 h oridonin treatment in different dose.

**Supplementary Figure 2:** Oridonin induce Caspase3 activation and PARP cleavage in p53 wildtype cancer cell RKO in dose-dependent manner. RKO cells were treated with oridonin in different dose for 24h, then caspase3 and PARP in cell lysates were detected by WB.

**Supplementary Figure 3:** Autophagy related protein beclin-1, Atg5 and LC3-II were unregulated under oridonin treatment in p53-mutated cell HCT-15, while such phenomenon were no detected in p53 wildtype cell HCT116. HCT-15 cells were treated with 15μM oridonin and HCT116 were treated with 30 oridonin for 24h, then then beclin-1, Atg5 and LC3 in cell lysates were detected by WB.

**Supplementary Figure 4:** Oridonin could induce LC3-II in xenograft model.
