# Supplementary figures and images for: Oridonin induces autophagy via inhibition of glucose metabolism in p53-mutated colorectal cancer cells
Source: Cell Death Dis. 2017 Feb 23;8(2):e2633–. doi: 10.1038/cddis.2017.35 (PMC5386482; doi:10.1038/cddis.2017.35)

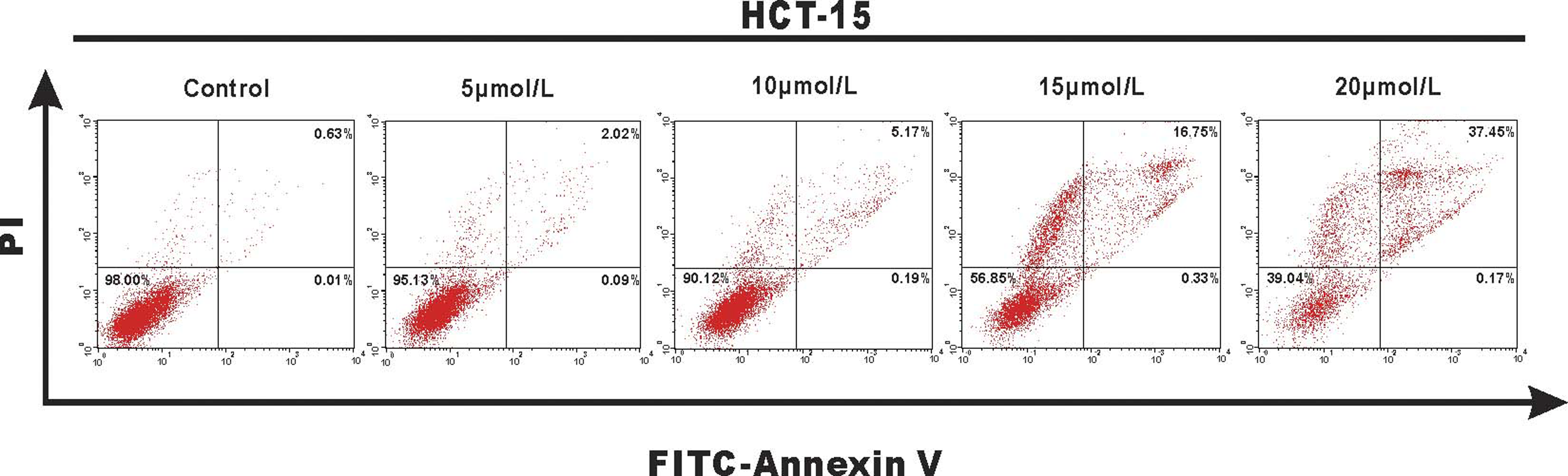

Supplement: Supplementary Figure 1 [file cddis201735x3.tif]

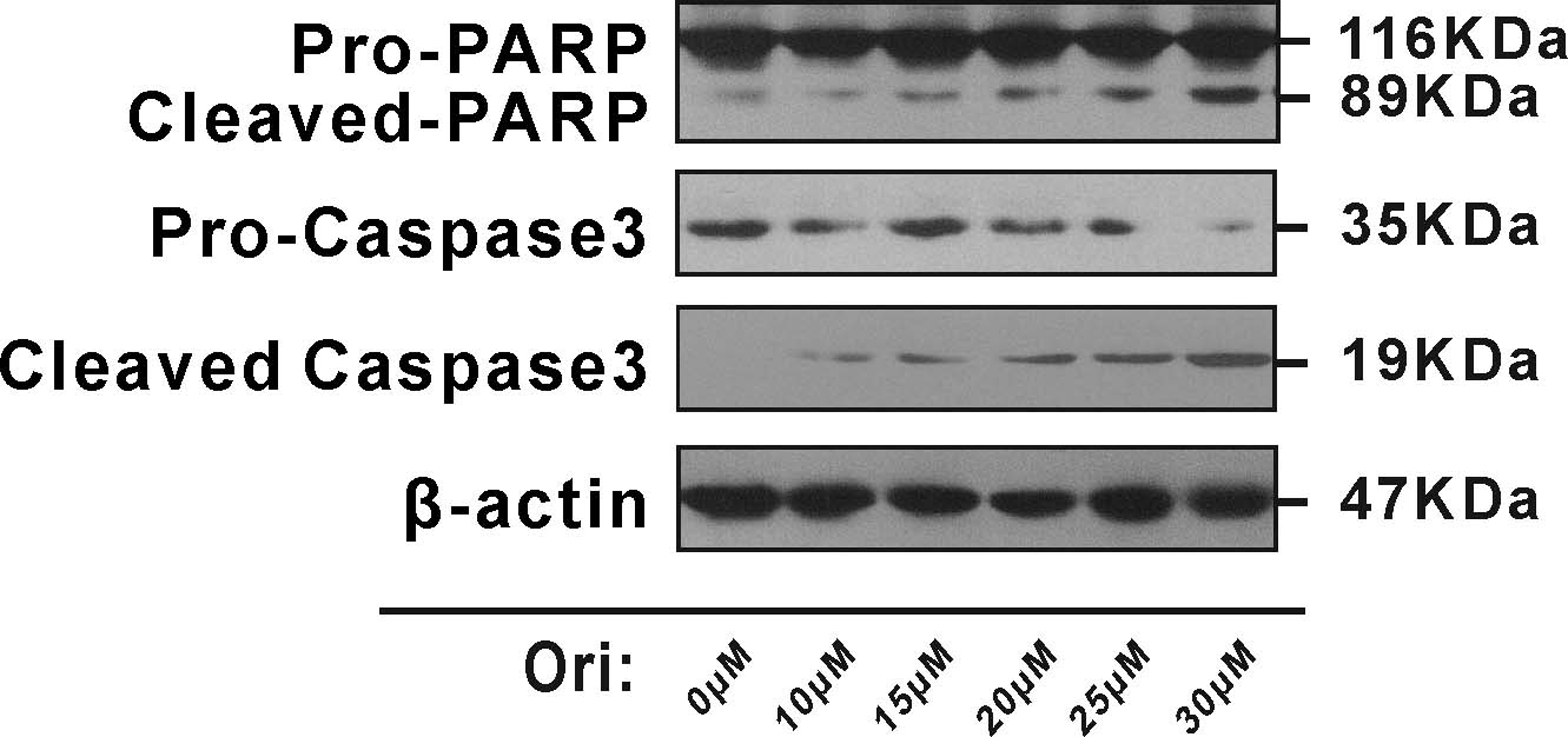

Supplement: Supplementary Figure 2 [file cddis201735x4.tif]

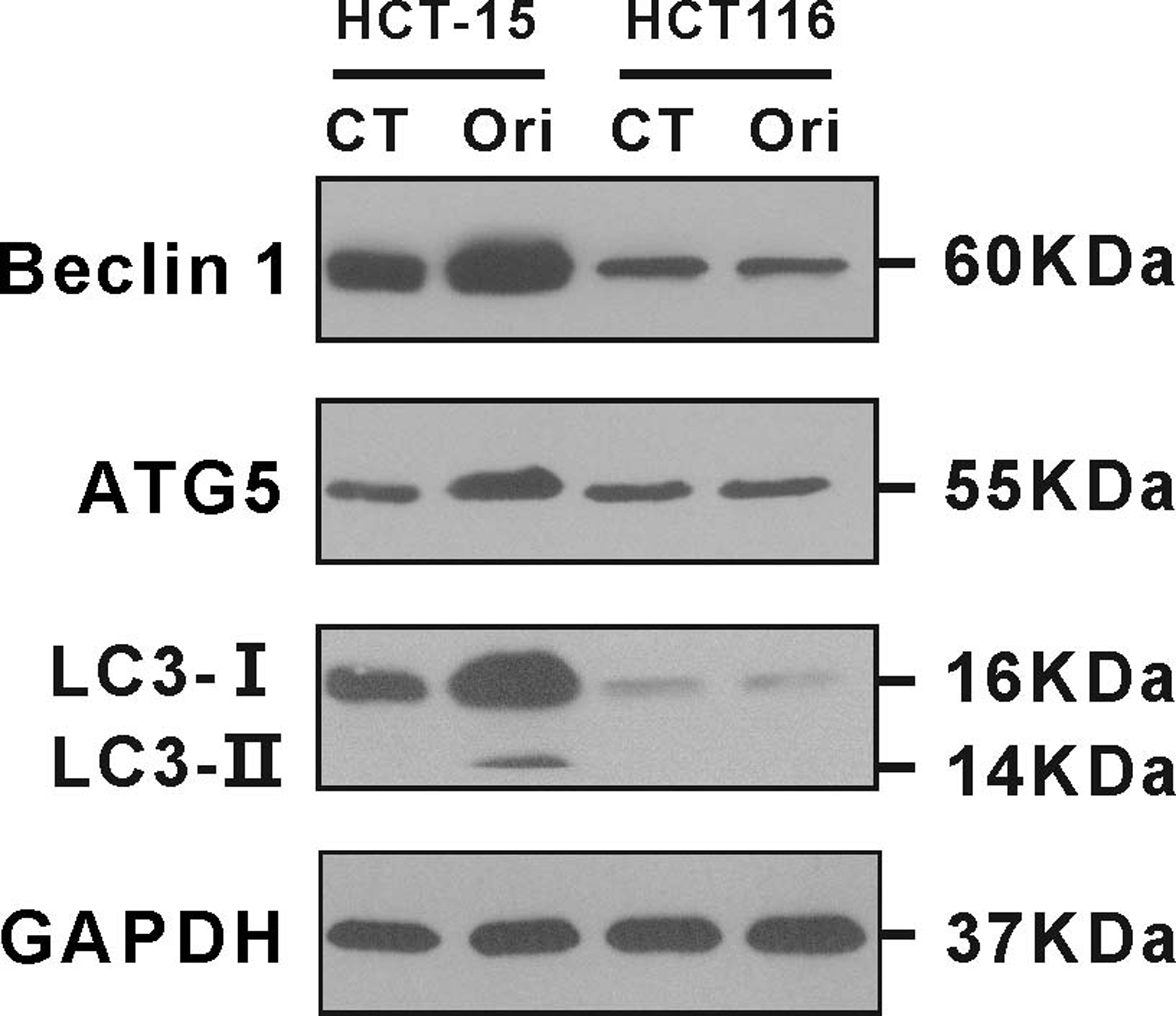

Supplement: Supplementary Figure 3 [file cddis201735x5.tif]

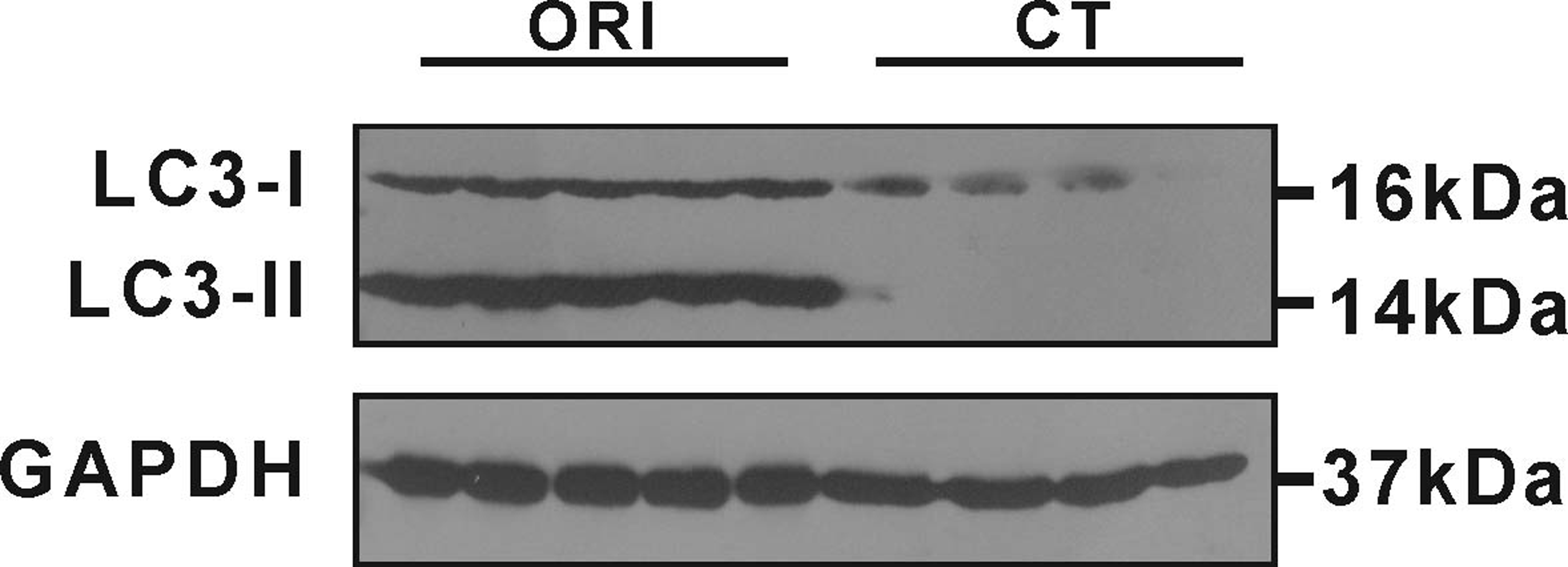

Supplement: Supplementary Figure 4 [file cddis201735x6.tif]
